# The Influence of Employee Emotion Fluctuation on Service Performance: An Experience Sampling Data Analysis

**DOI:** 10.3389/fpsyg.2022.648142

**Published:** 2022-02-21

**Authors:** Biqian Zhang, Lei Zhao, Xiaoyan Liu, Yinwei Bu, Yingwei Ren

**Affiliations:** ^1^School of Business Administration, Faculty of Business Administration, Southwestern University of Finance and Economics, Chengdu, China; ^2^Department of Regional Economics, West Center for Economic Research, Southwestern University of Finance and Economics, Chengdu, China; ^3^Department of Business Administration, Research Institute of Economics and Management, Southwestern University of Finance and Economics, Chengdu, China; ^4^Chengdu Branch, Industrial and Commercial Bank of China, Chengdu, China

**Keywords:** negative emotion, positive emotion, service performance, regulatory emotional self- efficacy (RESE), sleep quality

## Abstract

Research on the relationship between emotions and job performance is ubiquitous, yet few scholars have examined the combined effects of different emotions. Drawing on the broaden-and-build theory and conservation of resources (COR) theory, we propose that employees’ daily emotion fluctuations (positive emotions vs. negative emotions) will affect their service performance in opposite directions. Furthermore, we propose these effects will be moderated by psychological [i.e., regulatory emotional self-efficacy (RESE)] and physiological (i.e., sleep quality) characteristics of the employees. Based on the experience sampling method (ESM), data (*N* = 810) obtained from 187 frontline employees of 35 bank branches over 18 consecutive days supports our hypotheses.

## Introduction

The emotions shown by service workers can be directly connected with the profit of the service company (Han et al., 2018). The past decade therefore has observed significant advances in research of emotional experiences in workplace (e.g., Pugh, 2001; Totterdell and Niven, 2014; Ibrahim et al., 2016) and has established the powerful impacts of employee emotion on job performance (George, 1995; Williams and Shiaw, 1999; Furnham and Milner, 2013; Chu, 2016; Liu, 2016). In the service sector, frontline staff not only bridge the organization and the customers together but also play an essential role in firm differentiation and competitive advantages (Hui and Zhen, 2013). As a result, there has been a growing number of research studies conducted to understand factors that may lead to performance fluctuations in service delivery during the past decades (Parasuraman et al., 1985; Pugh, 2001; Lechner et al., 2020).

From the role expectation perspective, Rafaeli and Sutton (1987) theorized how emotion expression affects employees’ outputs and found that a warm emotional front promotes sales, particularly when customers expect that it should be a central part of a firm’s service. Along their footsteps, scholars have increasingly begun to associate employees’ emotions with their service performance and have identified various individual and contextual antecedents, such as emotional intelligence (EI) (Prentice and King, 2011; Chi and Chen, 2013; Hur et al., 2020; Vashdi et al., 2021) and leadership (Wu et al., 2020). For instance, humorous leadership can induce employees’ positive emotions and thus, enhance employees’ passion and service performance (Wu et al., 2020); supervisor incivility, however, leads to emotional exhaustion and reduces job performance (Cho et al., 2016). In addition, EI has found positively associated with employee service performance (Prentice and King, 2011).

Work is an emotional experience, and the expression of emotions as well as creation of feelings are expected parts of many service employees’ work roles (Hochschild, 1983; Pugh, 2001). As a consequence, when a job helps employees attain terminal values, or engage in activities doing good for others, positive emotions will be experienced (Totterdell et al., 2011; Disabato et al., 2017; Park et al., 2020). Following “primitive emotional contagion” (Hatfield et al., 1993), employees’ positive emotions can go beyond the individual level to influence one’s group interactions, such as group cooperation (Barsade, 2002; Barsade et al., 2018) and team creativity (Tang and Shu, 2011). Negative emotions that are inevitable in workplace, however, are detrimental to performance improvement, by inhibiting learning of complex structures (Shang et al., 2013), or shifting employees’ focus from factors that benefit performance improvement to those with a negative effect (Beal et al., 2005).

More importantly, the effect of emotions on service performance is immediate. The impressions for a few seconds made through emotions shown by service workers to customers in the process of service production can be directly connected with the service performance (Han et al., 2018). Considering the dynamic nature of emotions at work, a research method that can explore the relationship between emotion and service performance in a more timely and continuous manner will be valuable. Prior research has suggested the experience sampling method (ESM), i.e., repeated sampling of experiences in real time in natural contexts, as a more suitable method to understand how performance can be affected by temporary emotional state (Hormuth, 1986). Compared to traditional retrospective questionnaire survey method, ESM that repeatedly gathers experience and behavior data at random or pre-set times during the activity of interest can generate more accurate estimation of the variables and free researchers from reliance on participants’ recall (Alliger and Williams, 1993; Shiffman et al., 2008). ESM is even more suitable to study the temporary impacts of emotion as it not only measures the mood variance among individuals but also within individuals across time (Weiss et al., 1999; Ilies and Judge, 2004). As we all know that one’s mood on Monday morning will be very different from that on Friday afternoon (at intra-individual level) and such variation might be different for different people across the week (at inter-individual level) (Miner et al., 2005).

In light of the aforementioned advantages, ESM has been widely used to understand the impact of temporary emotion at work (Weiss et al., 1999; Weise, 2002; Ilies and Judge, 2004; Han et al., 2019). For example, Xu and Wang (2019) adopted experience sampling methodology to examine the effects of hotel employees’ emotion variation across 9 weeks, and found that higher variability in negative emotions predicted higher emotional exhaustion and lower job satisfaction. In addition, Han et al. (2019) conducted a study on the relationship between moods and creativity across 15 consecutive days, and found that positive moods were positively correlated with creativity, while negative moods were associated with lower creativity.

Research that applies ESM to explore how employees’ daily emotion fluctuations affect their service performance, however, is relatively scant. Addressing this research gap, over 18 consecutive working days, we sent repeated questionnaires to a total of 187 frontline employees from a large Chinese state-owned bank to measure their daily positive and negative emotion and perceived service performance. Results of hierarchical linear modeling analyses suggest that employee’s emotional state is highly correlated with their service outputs on a daily basis. Specifically, positive emotions predict better service performance while negative emotion impairs service performance, at both intra-individual and inter-individual levels. Moreover, for employees with higher regulatory emotional self-efficacy (RESE) and better sleep quality, the positive effect of positive emotion is strengthened while the negative effect of negative emotion is alleviated.

Our current research contributes to the existing literature on emotions and job performance in three meaningful ways. First, our work reveals the temporary and transient effect of emotion on service performance within individuals. Fisher (2002) found as much as 47% of the variance in positive momentary affective reactions and 77% in negative affective responses at work occurred within rather than between individuals. Similarly, Miner et al. (2005) revealed that 56% of the variance in hedonic tone of mood was within- rather than between-individual through experiments. The difference in individual emotions is worthy of deeper investigation. Our work, therefore, expands prior research by looking into how temporary emotion fluctuations of frontline staff affect their service performance every day.

Second, prior literature has categorized emotions as either positive or negative along a continuum and attended to only one of the two types of emotions (Shang et al., 2013; Rispens and Demerouti, 2016; Paakkanen et al., 2020), while rather limited research has examined both positive and negative emotions at the same time. We address the research gap by assessing the effects of both positive and negative moods at work.

Finally, prior research has mainly focused on the moderating roles of employees’ psychological characteristics (e.g., organizational commitment, professional identity, and job satisfaction), while less focusing on physiological factors, sleep quality in particular. Sleep deprivation plays an essential role in various cognitive and behavioral outcomes such as impaired working memory (Chuah et al., 2010), lower vigilance and longer reaction time (Franzen et al., 2008), increased hostility and fatigue (Scott and Judge, 2006), and consequently leads to lower efficiency and job performance (Diestel et al., 2015). Our work contributes to related literature by investigating how sleep quality might moderate the impacts of employees’ emotion fluctuations on their service performance.

## Theory and Hypotheses Development

### Positive Emotion and Service Performance

Frijda (1988) defined emotion as an active state produced by a mismatch between an organism’s goals and some internal or environmental stimulus. The leading approach in emotion psychology that addresses the structure of emotional experiences is rooted in the idea of core affect (e.g., the sense of pleasure, elation, and tension) and its circular structure (Russell, 1980; Feldman Barrett and Russell, 1998; Stanisławski et al., 2021). Watson and Tellegen (1985) proposed a two-factor structure that orthogonalizes positive affect and negative affect and formed octant to different degrees. More specifically, in their research, “pleasantness octant” contains items representing a mixture of high positive affect and low negative affect, while “unpleasantness octant” includes words with high negative affect and low positive affect. Different emotional experiences can leave various traces without any necessity to reconcile them (Abelson et al., 1982). When facing different situations, individuals might regulate their emotions consciously and non-consciously, and do something that they think is more appropriate under such context (Gross, 1999, Gross, 2015). Thus, emotions can be either helpful or harmful, depending on the context and timing (Gross, 2001).

Specifically, positive emotion is a unique immediate response to something meaningful and entails temporary pleasure. Fredrickson (2004) proposed the broaden-and-build theory that positive emotion is an essential element of optimal functioning and, therefore, a critical predictor of individual wellbeing. “Broaden” refers to the phenomenon that when individuals experience positive emotions in a nonthreatening situation, they tend to engage in nonspecific actions and become more focused and open (Fredrickson, 2004; Fredrickson and Branigan, 2005). Performance in tasks tapping divergent thinking (e.g., brain storming) can be reliably improved by inducing positive moods (Baas et al., 2008; Davis, 2009), as people experience innovative impulses with positive emotions, by (1) increasing the number of cognitive elements available for the association; (2) paying attention to broader elements relevant to the problem; and (3) enhancing cognitive flexibility (Isen et al., 1987). In this way, positive emotions expand the scope of an individual’s attention, cognition, and action. “Build” refers to the increase in an individual’s daily positive emotional experience, which can bolster a series of personal resources (e.g., physical, cognitive, psychological, and social resources) and promote happiness and personal growth. Specifically, positive emotions can increase individual psychological resources such as self-confidence and optimism, and thus enhance employees’ involvement in service work (Schaufeli and Bakker, 2004; Hui and Zhen, 2013). When employees experience positive emotions at work, they tend to interpret ambiguous information in self-benefitting ways, be more focused and patient when serving customers, and be more active in expanding work ideas and solving business problems (Tamir and Robinson, 2007; Johnson and Stapel, 2011). Therefore, we posit the following hypothesis:

H1. Employees’ positive emotions will positively predict perceived service performance.

### Negative Emotion and Service Performance

Negative emotions refer to short-term negative and unstable emotional states experienced by employees in the workplace (Fisher, 2002; Totterdell and Niven, 2014). According to the valence hypothesis, the right hemisphere of the brain is activated when an individual experiencing a negative emotion, which results in defensive and withdrawal behaviors (Ahern and Schwartz, 1985). From the perspective of biological evolution, negative emotions (e.g., anger, fear, and disgust) can motivate adaptive behaviors (e.g., fight, flight, and freeze responses) in the face of threat (Davidson et al., 2000). In the workplace, however, extensive research has verified that negative emotions hamper employees’ job performance. For example, anger narrows and restricts cognitive processing, by which the integration of the emotional route and the cognitive route hampers employees’ performance in complex tasks (Miron-Spektor and Rafaeli, 2009). In addition, exchanging threats during a negotiation inhibits creativity and flexible thinking, which results in less integrative agreements (De Dreu et al., 1998). According to the Conservation of Resources (COR) theory (Hobfoll, 1989), negative emotions can powerfully activate the negative information stored by employees and reduce their work enthusiasm. As evidence, passive moods reduce organizational citizenship behavior (OCB), while promoting counterproductive work behavior (CWB) in workplace (Sun et al., 2014). To compensate for their negative emotions, employees need to invest many psychological resources, leaving them limited mental resources to improve their service performance. Taken together, we propose:

**H2.** Employees’ negative emotions will negatively predict perceived service performance.

### The Moderating Role of Regulatory Emotional Self-Efficacy

As a growing emphasis on the generative, creative, proactive, and reflective properties of the human’s mind, social cognitive theorists have now attended to the role of self-efficacy beliefs in emotion-related self-regulation (McCraty et al., 1998; Heuven et al., 2006; Caprara et al., 2008). Bandura et al. (2003) defined RESE as individual’s confidence in whether one can effectively regulate his/her emotional states, including perceived ability to: recognize emotional states, understand other people’s feelings, and manage positive and negative emotional expressions. Caprara et al. (2008) further theorized RESE as a complex process of initiating, avoiding, inhibiting, maintaining, or modulating internal feelings and different emotion-related components in the service of individual adjustment. A similar concept to RESE is EI. Mayer and Salovey (1997) proposed the EI ability-based model, in which EI was defined as the ability to appraise, perceive, and express emotions rightly. It is the ability to comprehend emotion and, in the process, develop emotional knowledge. The difference between the two concepts is that RESE is a subjective concept regarding individual’s perception of his or her emotion-related abilities. EI, however, is not only perception but also understanding, regulation, and assimilation of emotions (Mayer and Salovey, 1997). In addition, RESE specifically focuses on the management process of positive and negative emotions.

Self-efficacy theory suggests that individuals’ belief that they cannot cope with dangerous events (i.e., low self-efficacy) is the main reason for anxiety, fear, and withdrawal behaviors. In the face of stress and provocation, individuals who are unable to fully and effectively adjust their strong negative emotions may either improperly materialize and magnify the negative emotions, or be overwhelmed by fear, anxiety, and depression, which all negatively affect their work performance (Waugh et al., 2008; Rothbard and Wilk, 2011). To the contrary, experiencing positive emotions can enhance one’s cognitive functions, relieve the disturbing emotions caused by awful experiences, and make it easier for people to obtain adaptive coping and positive social interaction experiences (Fredrickson and Branigan, 2005; Fredrickson et al., 2008). RESE includes both the abilities to manage negative (NEG) affect and express positive (POS) affect (Caprara et al., 2008). We use the cognitive-affective processing system (CAPS) model (Mischel and Shoda, 1995) to explain how RESE moderates the relationship between positive/negative emotions and service performance. Metcalfe and Mischel (1999) proposed that there are two types of processing—hot and cool—involving distinct interaction systems. The *cool cognitive system* is the seat of self-regulation and specializes in complex spatiotemporal and episodic representation and thought, which is also noted as the “know” system. The *hot emotion system* is fundamental for emotional (classical) conditioning and undermines efforts at self-control. It specializes in quick emotional processing and response based on unconditional or conditional trigger features, which is known as the “go” system (Metcalfe and Jacobs, 1998; Metcalfe and Mischel, 1999).

RESE not only stimulates employees’ efficacy beliefs in their ability to better express positive emotions such as happiness, excitement, and pride, but also prevents individuals from being knocked down by negative emotions such as anger, disappointment, and discouragement in front of adversity or frustrating events (Bandura, 1995; Bandura et al., 2003). Therefore, employees with high RESE can quickly and effectively shift from the *hot emotional processing system* to the *cool cognitive processing system* to achieve greater self-control and behavior regulation (Metcalfe and Jacobs, 1998).

Employees with high RESE can maintain or improve positive emotional behaviors and restrain negative emotional, impulsive behaviors (Bandura et al., 2003). Therefore, the role of positive emotions in improving service performance might be strengthened for high RESE employees, while the negative consequences of negative emotions will be weakened. However, for employees with low RESE, it is difficult for them to switch to the *cool cognitive processing system* when the *hot emotional processing system* is triggered. Consequently, positive emotions are less likely to generate positive behaviors or service improvement, while negative emotions may result in even worse service performance. Taken together, employee’s RESE can mitigate the disturbance of service performance by emotional fluctuations. Specifically, we posit the following hypotheses:

**H3a.** The effect of positive emotions on perceived service performance will be moderated by RESE, with the effect is stronger for employees with high (vs. low) RESE.
**H3b:** The effect of negative emotions on perceived service performance will be moderated by RESE, with the effect is weaker for employees with high (vs. low) RESE.

### The Moderating Role of Sleep Quality

Sleep quality refers to the duration of the feeling of tiredness, the sense of energy when awake during the day, and periods of sleeping and wakefulness at night (Harvey, 2008). Sleep may seem to be a state of inactivity, but it involves a host of physiological processes. It repairs mammalian bodies in a way that no other measures can. The amplitude of the changes in brain metabolism and neuronal activity that occur during sleep exceeds that of those that occur during most waking periods. The argument that sleep serves a vital function is compelling (Siegel, 2005). Optimal sleepers report fewer symptoms of depression and anxiety, but higher levels of environmental mastery, personal growth, positive relations with others, purpose in life, and self-acceptance (Hamilton et al., 2007). Individuals with adequate sleep or good sleep habits experience more positive emotions and fewer depressive symptoms (Hamilton et al., 2007).

Regarding job outcomes, lack of sleep decreases individuals’ self-control while increasing hostility and thus results in greater workplace deviance (Christian and Ellis, 2011). Furthermore, in a controlled experiment, Barnes et al. (2016) revealed that sleep deprivation harms the attribution of charismatic leadership for both leaders and followers, with leaders’ poorer emotional regulation and followers’ negative emotions work as the underlying mechanism, respectively. Therefore, insufficient sleep directly reduces the effectiveness of leadership and affects the leader-follower relationship.

To sum up, sleep quality is critical for both emotional regulation and job performance. High-quality sleep not only enables employees to maintain an optimistic work attitude toward customers but also releases cognitive depletion caused by negative emotions (Kühnel et al., 2018; Hisler et al., 2019). Therefore, we expect that, for employees with high sleep quality, the negative impact of emotion fluctuations on service performance will be attenuated. This is because high sleep quality will strengthen the positive effect of positive emotions on service performance while mitigating the adversity of negative emotions on service performance. In contrast, for employees with low sleep quality, the emotional fluctuation effect on service performance might even be exacerbated. Due to reduced biological support after sleep deprivation, employees are less likely to give their positive emotions a full play to improve their service performance and or even have to invest additional mental resources to work for a longer time. In addition, when experiencing negative emotions, low sleep quality will hamper employees’ ability to adjust and transform these negative emotions into positive ones.

Taken together, sleep quality can “iron out” the disturbance of service performance by emotion fluctuations at the individual level. We therefore posit the following hypotheses:

**H4a:** The effect of positive emotions on perceived service performance will be moderated by sleep quality, with the effect is stronger for employees with high (vs. low) sleep quality.
**H4b.** The effect of negative emotions on perceived service performance will be moderated by sleep quality, with the effect is weaker for employees with high (vs. low) sleep quality.

**FIGURE 1:**
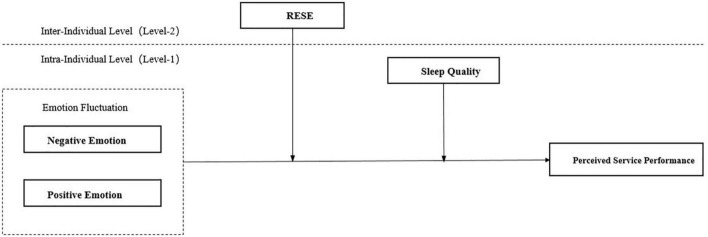
Theoretical research model.

Figure 1 describes our theoretical research framework.

## Materials and Methods

### Experience Sampling Method

We used the ESM for data collection and dynamic evaluation. The ESM is essentially a self-report method for data collection that lasts for a period of 1–2 weeks, during which the researchers send reminders to subjects every day, to answer questions at multiple moments marked by the occurrence of certain events or at random times (Hormuth, 1986; Hektner et al., 2006). Compared to traditional, one-wave questionnaire survey that heavily relies on participants’ (possibly biased) recall, this method has various advantages including: (a) it has high ecological validity and can be used to dynamically observe the actual states of the participants; (b) it is suitable for observing variations within-subjects and understanding what individuals experience, feel, think, and act in a more natural setting; and (c) it is suitable for improving the accuracy of longitudinal research via the collection of multiple measurements. Therefore, the ESM is currently one of the most appropriate methods to study the impacts of emotion fluctuations in individuals.

### Participants and Procedures

A total of 187 frontline employees from 35 branches of a large Chinese state-owned bank participated in our study. The online questionnaire system was used to gather survey data at two stages. At the first stage, subjects’ demographic information (i.e., gender, age, and education) and individual characteristics (RESE) were collected. Employees were required to provide their unique employee code in the banking system to facilitate follow-up data tracking and matching. Two weeks after the first stage, the second stage was conducted using the repeated ESM.

According to the work shift regulations of the bank, frontline employees followed either a 5-days-on and 2-days-off schedule or a 2-days-on and 1-day-off schedule. To ensure the validity of the questionnaire, we did not invite off-day employees to participate in the survey. In addition, to establish a reasonably long period for data collection, the second stage adopted a rolling survey spanning 18 consecutive days. With the consent of all participants, each was invited to complete two different questionnaires per working day. As one’s emotional state and sleep quality are less prone to interference in the morning, and one’s memory of these states has high fidelity at this time, subjects were required to complete the survey of positive emotion, negative emotion, and sleep quality between 7:00 a.m. and 9:00 a.m. every morning. Since employees’ job performance can be only determined after the work has been done for the day, participants were required to complete the perceived service performance survey between 5:00 p.m. and 8:00 p.m. in the afternoon. The survey was distributed to participants by the branch manager through WeChat (an instant communication APP widely used in China for both work and life). Participants only provided their unique employee code and then completed the questionnaire, which took approximately 2–3 min.

Over a period of 18 consecutive working days, we collected 1,001 questionnaires completed in the morning and 990 ones completed in the afternoon. Together, these yielded 187 inter-individual observations and 1,181 intra-individual observations. Matching each observation with the unique employee code, a total of 81 employees completed the two questionnaires for 10 consecutive working days. In the end, we obtained 81 inter-individual and 810 intra-individual data points for final analysis, and the percentage of valid data points was 43.32% for inter-individual observations and 68.59% for intra-individual observations. Of the 81 employees, 58.62% were females, 45.98% were 25–35 years old, 68.97% had a bachelor’s degree, and 44.82% had more than 15 years plus working experience.

### Measures

#### Positive Emotion

We measured employees’ positive emotion using the 9-item short-scale adapted from the Chinese version Positive and Negative Affect Schedule (PANAS; Qiu et al., 2008), with sample items such as “excited” and “enthusiastic.” This scale was revised and tested for use in the Chinese setting and has been proved to have good reliability and validity (Li et al., 2021; Pang and Wu, 2021; Yang et al., 2021). Participants responded to this scale on a 5-point Likert scale ranging from 1 (“strongly disagree”) to 5 (“strongly agree”). This scale had a Cronbach’s alpha value of 0.994 and an AVE value of 0.946, indicating robust reliability.

#### Negative Emotion

We measured employees’ negative emotion using the 9-item short-scale derived from the Chinese version PANAS scale (Qiu et al., 2008), with sample items such as “ashamed” and “distressed.” Participants completed this measure along a 5-point Likert scale ranging from 1 (“strongly disagree”) to 5 (“strongly agree”). The items had a Cronbach’s alpha value of 0.993 and an AVE value of 0.938, indicating robust reliability.

#### Perceived Service Performance

We measured employees’ perceived service performance using 10 items derived from the Bettencourt and Brown’s (1997) Service Behaviors Scale. The scale included sample statements such as “Voluntarily assists customers even if it means going beyond job requirements” and “Performs all those tasks for customers that are required of him/her.” Participants responded the scale on a 5-point Likert scale ranging from 1 (“strongly disagree”) to 5 (“strongly agree”). This measure had a Cronbach’s alpha value of 0.983 and an AVE value of 0.851, indicating robust reliability.

#### Sleep Quality

We measured employees’ sleep quality using a 4-item short-scale adapted from Jenkins et al.’s (1988) Sleep Quality Scale, with sample items such as “Have trouble falling asleep last night?” and “Wake up several times last night?” Participants responded to this measure on a 5-point Likert scale ranging from 1 (“strongly disagree”) to 5 (“strongly agree”). This measure had a Cronbach’s alpha value of 0.973 and an AVE value of 0.900, indicating robust reliability.

#### Regulatory Emotional Self-Efficacy

We measured employees’ RESE using the 12-item scale adapted from Caprara et al. (2008), with sample items such as “When something pleasant happens, I express my pleasure” and “When I achieve my goals, I feel good about myself.” Participants responded to this measure on a 5-point Likert scale ranging from 1 (“strongly disagree”) to 5 (“strongly agree”). This measure had a Cronbach’s alpha value of 0.986 and an AVE value of 0.855, indicating robust reliability.

### Statistical Analyses

As repeated measurements were carried out over 18 consecutive working days to form the panel data for intra-individual (positive emotion, negative emotion, sleep quality, and perceived service performance) and inter-individual (gender, age, education, and RESE) observations, we adopted the hierarchical linear modeling (HLM) random slope model processing method for the two-level analysis. The equations for the HLM analyses are as follows:


$$\text{Level-1 Model:}{\text{Y}}_{ij}=\beta_{0j}+\beta_{1j}\times (gender_{ij})+\beta_{2j}\times (age_{ij})+\beta_{3j}\times (education_{ij})+\beta_{4j}\times X_{ij}+r_{ij}$$

$$\text{Level-2 Model:}\beta_{0j}=\gamma_{00}+\gamma_{01}\times Z_{j}+{\text{μ}}_{0j}$$

β1j=γ1jβ2j=γ2jβ3j=γ3jβ4j=γ40+γ41×Zj+μ4j

Dependent variable Y is a continuous variable denoting perceived service performance.

Independent variable X is a continuous variable denoting positive emotion or negative emotion.

Independent variable Z is a continuous variable denoting RESE or sleep quality.

## Results

### Descriptive Statistics

Table 1 summarizes the means, standard deviations, and correlations of all the variables. At the intra-individual level, positive emotion was positively correlated with perceived service performance (*r* = 0.726, *p* < 0.01), while negative emotion was negatively correlated with perceived service performance (*r* = −0.648, *p* < 0.01). At the inter-individual level, the same pattern was observed, with a positive correlation between positive emotion and perceived service performance (*r* = 0.380, *p* < 0.01) and a negative correlation between negative emotion and perceived service performance (*r* = −0.334, *p* < 0.01).

**TABLE 1:** Descriptive statistics for the model variables.

Variables	*M*	*SD*	1	2	3	4	5	6	7
**Intra-individual variables (*N* = 810)**									
1 Service performance	3.912	0.786	1	0.380***	−0.334***				
2 Positive emotion	3.793	0.917	0.726***	1	−0.158**				
3 Negative emotion	2.035	0.905	−0.648***	−0.483***	1				
4 Sleep quality	3.373	1.067	0.392***	0.462***	−0.297***	1			
**Inter-individual variables (*N* = 81)**									
5 RESE	3.870	0.829	0.651***	0.577***	−0.611***	0.407***	1		
6 Gender	0.444	0.497	–0.021	–0.036	–0.020	–0.010	−0.092***	1	
7 Age	3.272	1.793	0.121***	0.136***	−0.066*	0.109***	0.139***	0.197***	1
8 Education	2.790	0.538	−0.196***	−0.175***	0.037	−0.122***	−0.151***	0.026	−0.453***

*p < 0.1, **p < 0.05, ***p < 0.01.

***Correlations above the diagonal**: service performance, positive emotion, negative emotion, and sleep quality were aggregated at the inter-individual level, and the correlations were then calculated together with RESE, gender, age, and education (N = 81).*

***Correlations below the diagonal**: Correlations below the diagonal represent the covariation among variables calculated from an independent variable’s HLM model with standardized intra-individual variables (Rothbard and Wilk, 2011).*

### Confirmatory Factor Analysis

We conducted a multilevel confirmatory factor analysis (MLCFA) using AMOS V25.0 to further test the extent to which daily positive emotion, daily negative emotion, daily sleep quality, daily perceived service performance, and individual RESE can be discriminated from each other. The results showed in Table 2 revealed that the five-factor model fitted the data best, indicating that the five variables in this study represent different constructs and can be empirically distinguished.

**TABLE 2:** Confirmatory factor analysis.

Models	Variables	χ^2^	df	χ^2^/df	CFI	RMSEA	SRMR
Five-factor	Service performance, positive emotion, negative emotion, sleep quality, RESE	2063.337	717	2.878	0.984	0.048	0.028
Four-factor	Service performance, positive emotion, negative emotion, sleep quality +RESE	2192.789	721	3.041	0.982	0.050	0.055
Four-factor	Service performance, positive emotion + negative emotion, sleep quality, RESE	2075.806	629	3.300	0.982	0.053	0.129
Three-factor	Service performance, positive emotion + negative emotion + RESE, sleep quality	1870.577	520	3.597	0.984	0.057	0.102
Three-factor	Service performance, positive emotion + negative emotion + sleep quality, RESE	2121.969	622	3.412	0.982	0.055	0.131
Two-factor	Service performance, positive emotion + negative emotion + sleep quality +RESE	1712.753	466	3.675	0.985	0.057	0.093
One-factor	Service performance+ positive emotion + negative emotion + sleep quality +RESE	1586.795	464	3.420	0.986	0.055	0.041

### Null Model Estimation

We used STATA 15.0 to examine the research model. To observe the variance of the intra-individual variables, we ran three null models (positive emotion, negative emotion, and perceived service performance) before the HLM analysis. The ICCs were 0.70 (or 70.03% of the variance) for perceived service performance, 0.74 (or 73.65% of the variance) for positive emotion, and 0.67 (or 67.07% of the variance) for negative emotion, confirming that the observations were not independent and justifying the use of HLM.

To minimize concerns regarding overlap among scales derived from common methods, we also conducted exploratory factor analyses on all scales constructed from the survey data. For negative emotion, positive emotion, RESE, sleep quality, and perceived service performance, a five-factor solution had all 9 negative emotion items loaded (all above 0.90) on the first factor, all 9 positive emotion items loaded (all above 0.88) on the second factor, all 12 RESE items loaded (all above 0.74) on the third factor, all 4 sleep quality items loaded (all above 0.80) on the fourth factor, and all 10 perceived service performance items loaded (all above 0.71) on the fifth factor. No cross loading problems was observed for the survey data, and thus addressing the common method variance concerns (Pugh, 2001).

### Hypotheses Testing

The hypotheses were developed at different levels. Negative emotion, positive emotion, sleep quality, and perceived service performance were measured at intra-individual level (level 1), while RESE, gender, age, and education were measured at inter-individual level (level 2). According to Enders and Tofighi (2007), centering within cluster (CWC) is appropriate if the Level 1 association between X and Y is of substantive interest, and CWC is preferable for examining cross-level interactions and interactions that involve a pair of Level 1 variables. Therefore, we used CWC method to process variables (negative emotion, positive emotion, sleep quality, and perceived service performance) and tested our hypotheses at the intra-individual level.

The main effect of positive/negative emotions on perceived service performance and the moderating effects of RESE and sleep quality, at both the intra-individual and inter-individual levels, were summarized in Tables 3, 4. Specifically, on a daily basis, the results of multilevel analyses in M2 showed that positive emotion was positively related to employees’ perceived service performance (γ = 0.471, *p* < 0.01), while results of M4 revealed that negative emotion was negatively associated with perceived service performance (γ = −0.334, *p* < 0.01). In line with our predictions and prior research, results suggested that positive emotions resulted in higher perceived service performance while negative emotions led to poor service performance of the frontline employees. Thus, H1 and H2 were both supported.

**TABLE 3:** Multilevel analyses: Perceived service performance by daily emotion fluctuations and RESE.

Variables	Perceived service performance (*N* = 810)				
	Positive emotion			Negative emotion	
	Null model M1	Main effects model M2	Regulatory effect model M3	Main effects model M4	Regulatory effect model M5
**Intra-individual variables (*N* = 810)**					
Intercept	3.912***	4.036***	4.057***	4.675***	4.511***
Positive emotion		0.471***	0.362***		
Negative emotion				−0.334***	−0.224***
**Inter-individual variables (*N* = 81)**					
RESE			0.397**		0.384***
Gender		0.010	0.073	–0.101	–0.064
Age		0.025	0.0014	0.005	–0.007
Education		–0.092	–0.085	–0.251	−0.159*
Positive emotion * RESE			0.117**		
Negative emotion * RESE					0.220***
*σ^2^*	0.617	0.138	0.140	0.146	0.105
*τ_00_*	0.436***	0.116***	0.064***	0.180***	0.136***
*τ_11_*		0.093***	0.057***	0.155***	0.095***

*p < 0.1, **p < 0.05, ***p < 0.01.

*The HLM stochastic slope model was used. Total centralization with positive emotion, negative emotion (Level-1), and RESE (Level-2). The results were reported with robust standard errors.*

**TABLE 4:** Multilevel analyses: Perceived service performance by daily emotion fluctuations and sleep quality.

Variables	Perceived service performance (*N* = 810)				
	Positive emotion			Negative emotion	
	Null model M6	Main effects model M7	Regulatory effect model M8	Main effects model M9	Regulatory effect model M10
Intercept	4.575***	4.24***	4.196***	4.608***	4.594***
Positive emotion		0.613***	0.573***		
Negative emotion				−0.557***	−0.505***
Sleep quality			0.021		0.174***
Positive emotion * sleep quality			0.086***		
Negative emotion * sleep quality					0.065**
Gender	–0.041	0.015	–0.011	–0.05	–0.036
Age	0.021	–0.006	–0.004	0.004	–0.006
Education	−0.255***	−0.113***	−0.109***	−0.246***	−0.225***
*R* ^2^	0.040	0.533	0.544	0.450	0.490
Adjusted *R*^2^	0.037	0.530	0.540	0.448	0.486
F	*F*(3, 806) = 11.337	*F*(4, 805) = 229.328	*F*(6, 803) = 159.584	*F*(4, 805) = 164.950	*F*(6, 803) = 128.577

***p < 0.01 and **p < 0.05.

*The HLM stochastic slope model was used. Total centralization with positive emotion, negative emotion (Level-1), and RESE (Level-2). The results were reported with robust standard errors.*

To observe in greater depth about how positive emotions and negative emotions dynamically affect perceived service performance, we analyzed the correlation between emotional fluctuation and perceived service performance. We computed employees’ emotion fluctuation by subtracting the negative emotion from the positive emotion. As expected, the regression result showed that (positive) emotion fluctuation was positively associated with perceived service performance (γ = 0.111, *p* < 0.01).

H3a stated that RESE moderates the relationship between employees’ positive emotions and perceived service performance. As we reported in M3, the main effect of RESE was significant and positive (γ = 0.397, *p* < 0.05), indicating that employees’ belief in their ability to regulate emotion would help to improve their perceived service performance in general. More importantly, the interaction between positive emotion and RESE was significant (γ = 0.117, *p* < 0.05). Since positive emotion showed a positive impact on perceived service performance (γ = 0.362, *p* < 0.01), it suggested that RESE further strengthened the positive effect of positive emotion.

H3b stated that RESE moderates the relationship between negative emotion and perceived service performance. The results of M5 first reconfirmed the positive relationship between RESE and perceived service performance (γ = 0.384, *p* < 0.05). It was further showed that the interaction term between negative emotion and RESE was significant and positive (γ = 0.220, *p* < 0.01). Considering the negative relationship between negative emotion and perceived service performance (γ = −0.505, *p* < 0.01), employees’ high RESE would help to mitigate the adverse effect of negative emotion on their daily performance.

H4a stated that sleep quality moderates the relationship between positive emotions and perceived service performance. According to M8, though sleep quality showed a null effect on employees’ perceived service performance (γ = 0.021, *p* > 0.1), its interaction with positive emotion on perceived service performance was significant and positive (γ = 0.086, *p* < 0.01). Specifically, high (vs. low) sleep quality enhanced the positive effect of positive emotion on employee perceived service performance.

H4b stated that sleep quality moderates the relationship between negative emotion and perceived service performance. In M10, we first found a positive effect of sleep quality (γ = 0.174, *p* < 0.01), suggesting that better sleep quality benefits employee perceived service performance in general. More importantly, the interaction term between negative emotion and sleep quality was significant (γ = 0.065, *p* < 0.01). Consistent with our prediction, a night of good sleep will help service employees regulate the negative emotions and thus mitigate the adverse effect of negative emotions on their performance.

**FIGURE 2:**
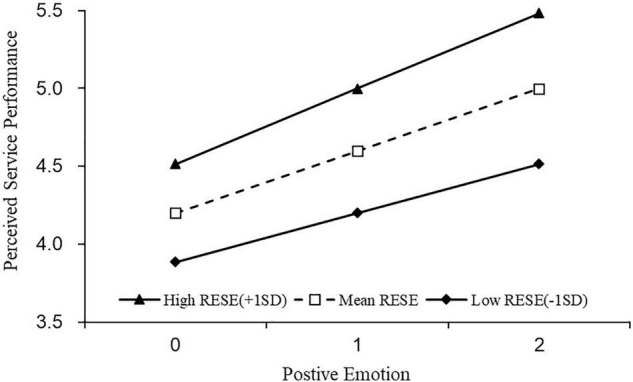
The moderating effect of RESE on the relationship between positive emotions and perceived service performance.

**FIGURE 3:**
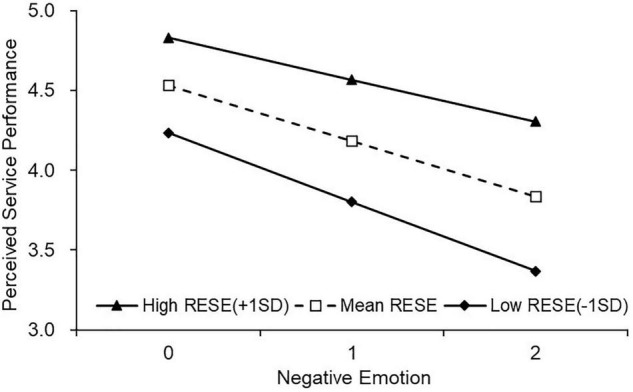
The moderating effect of RESE on the relationship between negative emotions and perceived service performance.

**FIGURE 4:**
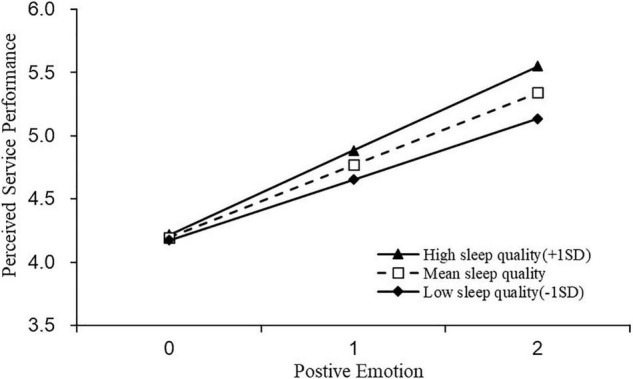
The moderating effect of sleep quality on the relationship between positive emotions and perceived service performance.

**FIGURE 5:**
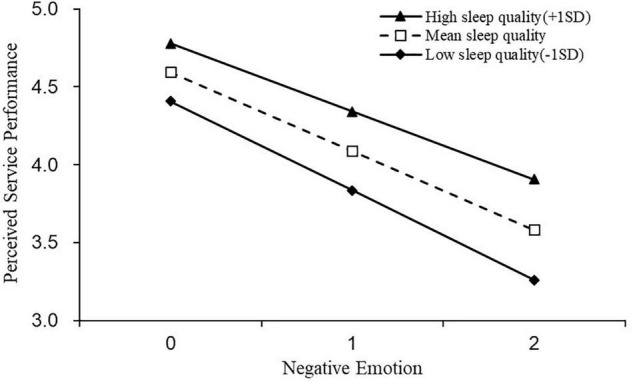
The moderating effect of sleep quality on the relationship between negative emotions and perceived service performance.

Simple slope tests for hierarchal linear models were used to examine whether the slopes in cross-level interactions were significantly different from zero (Preacher et al., 2006). The results are presented in Figures 2**–**5.

To further decompose the moderating effect of RESE, we first split the participants into employees with low RESE (−1*SD*; *N* = 110) or high (+1*SD*; *N* = 110), based on one standard deviation above or below the mean (Cohen and Cohen, 1983). Then we conducted a simple slop analysis to examine any difference in the impacts of positive and negative emotions on perceived service performance between high and low RESE employees. First of all, we observed a positive relationship between positive emotion and perceived service performance, for both low RESE (*b* = 0.314, *t* = 7.609, *p* < 0.01 and high RESE employees (*b* = 0.485, *t* = 19.131, *p* < 0.01), but the effect was significantly higher for high RESEs than that for low RESEs (*b_*high*_* = 0.485, 95% *CI* = [0.435, 0.534] vs. *b_*low*_* = 0.314, 95% *CI* = [0.233, 0.395]) (see Figure 2). Moreover, for employees with low RESE, negative emotion predicted lower perceived service performance (*b* = −0.434, *t* = −12.648, *p* < 0.01). Though negative emotion also impeded the perceived service performance of employees with high RESE (*b* = −0.263, *t* = −7.190, *p* < 0.01), the adverse impact was much weaker than that for low RESE employees (*b_*high*_* = −0.263, 95% *CI* = [−0.335, −0.191] vs. *b_*low*_* = −0.434, 95% *CI* = [−0.501, −0.367]) (see Figure 3). Thus, H3a and 3b were further supported.

Similarly, we grouped participants into employees with low sleep quality (−1*SD*; *N* = 174) or high sleep quality (+1SD; *N* = 186. We observed similar positive relationship between positive emotion and perceived service performance, across employees with low (*b* = 0.481, *t* = 12.964, *p* < 0.01) and or high sleep quality (*b* = 0.665, *t* = 20.887, *p* < 0.01), but the relationship was stronger for employees with high (vs. low) sleep quality (bhigh = 0.665, 95% *CI* = [0.603, 0.728] vs. blow = 0.481, 95% *CI* = [0.408, 0.553]) (see Figure 4). To the contrary, negative emotion led to lower perceived service performance for employees with both low (*b* = −0.574, *t* = −15.851, *p* < 0.01) and high sleep quality. For employees with high sleep quality (*b* = −0.436, *t* = −11.720, *p* < 0.01), but the influence was stronger for employees with low (vs. high) sleep quality (*b_*high*_* = −0.436, 95% *CI* = [−0.509, −0.363] vs. *b_*low*_* = −0.574, 95% *CI* = [−0.645, −0.503]) (see Figure 5). H4a and 4b were thus reconfirmed.

## Discussion

In this study, we investigated the relationships between emotion fluctuation and perceived service performance using experience sampling data analysis methods. The **ESM** was adopted in this research to conduct a dynamic rolling survey on frontline service employees over 18 consecutive working days. Compared with the cross-sectional data used in previous research, the time-series data used in our paper provided more details describing the emotion fluctuations of employees, i.e., both positive and negative emotions experienced every day. Our results revealed a positive correlation between individuals’ positive emotion and their perceived service performance. This aligns with conclusions in previous studies regarding positive emotion-work outcomes correlations: positive emotion is particularly likely to foster job satisfaction (Brief et al., 1995), work engagement (McGrath et al., 2017), work performance (Tenney et al., 2016), and team resilience (Meneghel et al., 2016). We also verified that negative emotions adversely affect service employees’ daily perceived service performance. This is consistent with previous studies on the correlation between negative emotions and work results: negative emotions are closely related to job stress and job strain (Brief et al., 1988), job burnout (Liu et al., 2021), and CWB (Sun et al., 2014).

We also identified two psychological and physiological variables, i.e., RESE and sleep quality, as the boundary conditions that moderated the effect of emotion on job performance. The moderating roles of RESE and sleep quality were confirmed by both regression analyses and simple slope tests. As an individual characteristic (Bandura et al., 2003), RESE can strengthen the positive effect of positive emotions while attenuating the negative effect of negative emotions on perceived service performance. Expanding prior research, we further verified that sleep quality moderated the impact of emotional experience on perceived service performance the next day (Alotaibi et al., 2020; Penggalih et al., 2021), with employees with better (vs. worse) sleep quality reported higher perceived service performance.

### Theoretical Contributions

Our research made two critical theoretical contributions. We first expanded the scope of the emotion-work relationship research. Prior research has mainly focused on either positive or negative emotional states. However, emotion is brief, intense, and highly volatile (Frijda, 1988). Therefore, perceived service performance under different emotional states needs to be measured in the shortest possible time frame to observe a specific emotion’s comparative effects more accurately. Based on the ESM, our study relied on daily observations of the frontline employees over 18 days. By measuring the emotional state and perceived service performance every day within the period, we obtained sufficient and reliable data to examine the temporary effects of both positive and negative emotions on perceived service performance more accurately. A more comprehensive investigation into the role of emotion fluctuations expands the existing literature on the emotion-work relationship.

We further explained how psychological and physiological factors moderated the effect of different emotional experiences on perceived service performance. The exploration of moderating factors in emotional management scenarios has focused on psychological traits (e.g., RESE). However, with more recent research in related fields using brain functional imaging technology, physiological factors such as sleep quality have been identified as critical predictors of employee emotion and work performance. Therefore, except from RESE, this study tested sleep quality as another moderator and confirmed the short-term moderating effect of sleep quality and the long-term moderating effect of RESE. These findings thus enriched the research on emotion-performance interactions.

### Limitations

Participants’ positive emotions, negative emotions, and sleep quality were all self-reported using the same questionnaire each morning, which may result in homologous errors, i.e., perceived service performance may be altered by the self-evaluation criteria. In future research, other sources of data can be obtained from the superiors and colleagues or via real performance indicators to reduce homologous errors and the potential influences of social approval. In addition, positive emotions (negative emotions) may have autocorrelation, and the perceived service performance level of the previous day might also affect the emotional state of the next day. Researchers in the future therefore could use wearable physiological monitors (Gabriel et al., 2019) to measure employees’ emotion states more accurately and more frequently.

Based on COR theory (Hobfoll, 1989), this study primarily focused on individual employees without considering the interactions between employees. Emotional contagion theory holds that one person’s emotional experience can stimulate the others (Hatfield et al., 1993). Prior literature on emotional contagion between employees and customers has mainly focused on the transference of emotions from employees to customers, while limited research has investigated emotional contagion between employees. Future research thus can explore how and when employees’ positive/negative emotions will be transferred to their colleagues and eventually affect employees’ service outputs.

Our current work has examined how RESE and sleep quality can regulate the relationship between positive and negative emotions on employee perceived service performance. Future studies can further explore how other factors such as personality characteristics, dialectical thinking, and EI may moderate the impacts of emotions on perceived service performance to have a more complete understanding of their relationships.

### Practical Implications

The findings of this study have valuable managerial implications as well. The service industry is characterized by intense competition, fast pace, high responsibility, high mechanical repetition, and stressful daily workload (Pugh, 2001; Lechner et al., 2020). For example, the Chinese National Mental Health Development Report (2017–2018) reveal that 48% of bank employees worked more than 50 h per week, and a large proportion of employees suffered from mental problems such as anxiety (54.9%), job burnout (30.6%), and depression (21.5%). Therefore, managers in the service industry need to pay greater attention to employees’ daily emotional states and behaviors and build a healthy work environment that can inspire positive emotions and alleviate negative emotions. This will ensure that frontline employees can maintain a good emotional state for long work hours.

Our work also suggests two strategies that managers can apply to enhance the benefits of positive emotions and reduce the harms of negative emotions on employees’ service performance. First, managers can improve employees’ RESE by offering regular training sessions on emotion control, stress management, and mindfulness. With improvement in ability for emotion regulation, the service employees will manage to improve their perceived service performance and suffer less from negative emotions (Waugh et al., 2008). Second, managers could optimize the administrative process by using an information management system or other methods to provide more flexible working schedules and avoid extensive overtime (Scott and Judge, 2006), and thus reducing insomnia and promote recovery following exertion at work (Sonnentag, 2003).

## Conclusion

This study explored the relationship between frontline employees’ emotion fluctuations and their (perceived) service performance and verified the regulatory effects of RESE and sleep quality from both psychological and physiological perspectives. Based on an ESM, and data collection from 187 bank frontline employees over 18 consecutive working days, we showed that positive emotions improve employees’ daily perceived service performance, as predicted by the broaden-and-build mechanism; in contrast, negative emotions hindered frontline employees’ daily perceived service performance due to the loss of psychological resources. Moreover, we showed that high RESE strengthened the positive relationship between positive emotions and perceived service performance while mitigating the negative relationship between negative emotions and perceived service performance. Finally, sleep quality moderated the relationship between emotions and service outputs in the same way as RESE.

## Data Availability Statement

The raw data supporting the conclusions of this article will be made available by the authors, without undue reservation.

## Ethics Statement

The studies involving human participants were reviewed and approved by the Southwestern University of Finance and Economics. The patients/participants provided their written informed consent to participate in this study.

## Author Contributions

BZ, YR, and YB conceptualized and designed the study. YB and BZ conducted the experiment. LZ and BZ performed the statistical analysis. BZ wrote the first draft of the manuscript. XL participated in manuscript writing and revision. All authors contributed to the article and approved the submitted version.

## Conflict of Interest

YB was employed by the company Industrial and Commercial Bank of China. The remaining authors declare that the research was conducted in the absence of any commercial or financial relationships that could be construed as a potential conflict of interest.

## Publisher’s Note

All claims expressed in this article are solely those of the authors and do not necessarily represent those of their affiliated organizations, or those of the publisher, the editors and the reviewers. Any product that may be evaluated in this article, or claim that may be made by its manufacturer, is not guaranteed or endorsed by the publisher.
